# The regeneration of *Acer rubrum* L. “October Glory” through embryonic callus

**DOI:** 10.1186/s12870-020-02496-z

**Published:** 2020-07-02

**Authors:** Chong-wen Dai, Yang-yang Yan, Yu-min Liu, Ya-min Liu, Yuan-wei Deng, Hong-yu Yao

**Affiliations:** 1 Resources and Environment Department of Southwest University, Three Gorges Reservoir area laboratory of ecological environment, Ministry of Education, Southwest University, Chongqing, 400715 China; 2 Institute of Mountain Hazards and Environment, Chengdu, Sichuan China

**Keywords:** *Acer rubrum* L. “October Glory “, Tissue culture, Embryogenic callus, Regeneration

## Abstract

**Background:**

Tissue culture and rapid propagation technology is an important way to solve the difficulties of plant propagation. This experiment aims to explore the appropriate conditions at each stage of the red maple’s tissue culture process and to obtain plantlets, thus providing a theoretical basis for the establishment of the red maple’s tissue culture system.

**Results:**

The results showed that the stem segment is the most suitable explant for inducing embryogenic callus. The MS (Murashige&Skoog) + 0.8 mg/L TDZ (Thidiazuron) + 1.0 mg/L 6-BA (6-Benzylaminopurine) + 0.5 mg/L IAA(Indole-3-acetic acid) + 35 g/L sucrose+ 7.5 g/L semi-fixed medium was the best for callus formation. When selecting type VI callus as embryonic callus induction material, MS + 0.6 mg/L TDZ + 0.5 mg/L 6-BA + 2.0 mg/L IAA + 35 g/L sucrose+ 7.5 g/L semi-fixed medium can get embryonic callus. The optimal medium for adventitious bud induction is MS + 1.0 mg/L TDZ + 3.0 mg/L 6-BA+ 0.2 mg/L NAA (1-Naphthaleneacetic acid) + 1.2 mg/L IAA + 35 g/L sucrose+ 7.5 g/L semi-fixed medium. The induction rate of adventitious roots in MS + 0.6 mg/L TDZ + 1.0 mg/L 6-BA+ 3 mg/L NAA + 35 g/L sucrose+ 7.5 g/L semi-fixed medium was the highest, reaching 76%.

**Conclusions:**

In the course of our research, we found that PGRs play an important role in the callus induction stage, and the effect of TDZ is particularly obvious; The callus cells grow and proliferate according to the “S” growth curve, and can be sub-cultured when the highest growth point is reached to maintain the rapid proliferation of the callus cells and to avoid inactivation of callus caused by tight niche.

## Background

Landscape industry has been recognized as the “eternal sunrise industry”. As a kind of ideal colored-leaf tree species, red maple has a promising market and is one of the most popular trees in modern landscape design planning in China [1]. However, due to environmental factors, the introduction of colored-leaf tree species is often accompanied by the problem of unstable leaf color and variation of progeny traits.

Introduced in China in 2011, October Glory (*Acer rubrum* L., red maple), originating in the northeastern United States, is an excellent cultivar, due to its beauty, colorful leaves, wet resistance, cold resistance, drought tolerance [2, 3], ecological characteristics [4, 5], and broad ability to adapt to a variety of soil types. After domesticating and cultivating the red maple, we selected this cultivar as it is well adapted to the climate conditions in southwest China (especially the bud mutations we selected) and has rapid growth along with stable leaf color. However, its low seed maturation rate and unstable leaf color traits of its progeny make it difficult to promote planting through seed propagation. The use of cuttings and other vegetative propagation methods are limited by the season and quantity of material. In addition, it is difficult to satisfy mass production requirements by small-scale cutting propagation. Therefore, it is of practical significance to establish the rapid propagation system of red maple.

Plant transformation regeneration systems mainly depend on whether they can form mature embryonic cells [6]. Therefore, the formation of callus embryo becomes the most important link. Types of explants, osmotic pressure, plant growth regulators (PGRs), environmental conditions, these factors play a vital role in the formation and maturation of embryonic callus [7–9]. Among them, the interaction between the medium and the PGRs is the basis for the establishment of embryonic cells [10, 11]. When a PGR stimulates explants and calluses, the internal intrinsic hormones produce feedback [12] and induce a callus to form an embryonic callus [13, 14]. In many cases, specific combinations and proportions of auxin and cytokinin can effectively induce embryonic callus or directional differentiation of organs [15, 16]. Somatic embryogenesis technology plays an important role in the preparation of artificial seeds and the genetic improvement of garden plants. This technology has been widely used in the breeding of garden plants that propagate slowly or have problems with propagation, and are of great significance for the propagation and conservation of rare plants [17, 18]. At present, the tissue culture and rapid propagation system of red maple is still being perfected, and yet there are no reports about red maple’s embryonic callus and adventitious organ formation.

To provide a theoretical basis and technical support for the production of *Acer rubrum* L. October Glory, this experiment explores the influence of culture medium and plant growth-regulating substances on callus induction in the procession of embryogenesis and plantlet regeneration and screened out the appropriate medium formulas in different stages.

## Results

### Callus induction

**Fig. 1:**
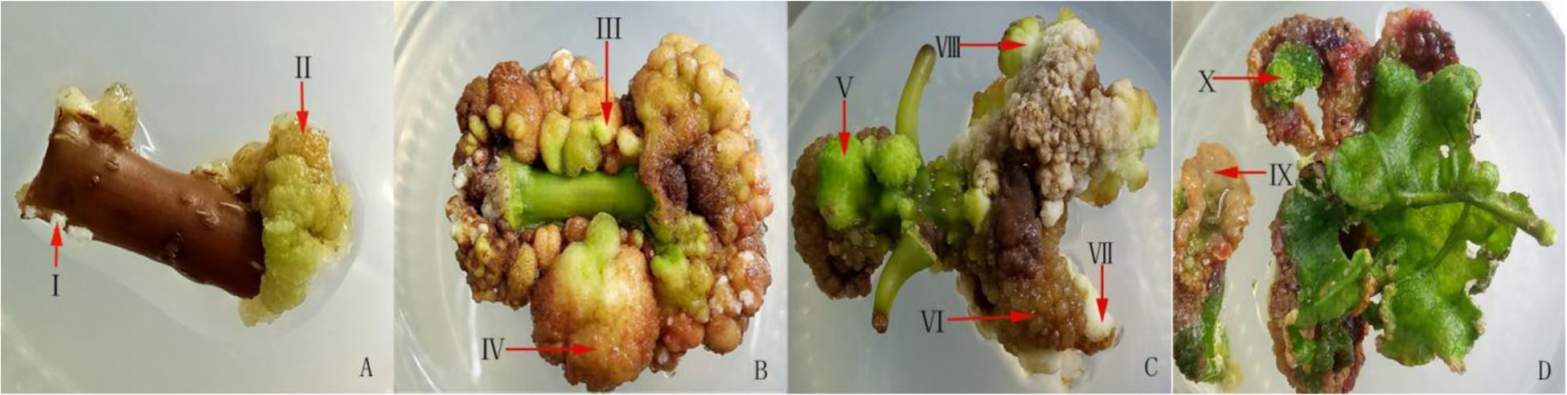
Callus of *Acer rubrum* L*.* explants in different periods **a**: Cultured 15 d, In the incision site and lenticel, explants produce I, II callus; **b**: Cultured 30 d, explant calluses were III and IV callus; **c**: Cultured 30 d, the stem section with a bud V, VI, VII and mixed type VII callus; **d**: Leaf explant culture for 25 d, IX and X calluses

During the period of callus induction, the callus induced by different types of explants showed significant differences. With the newly germinated twigs (approximately 15 d after germination, without buds) as the explants and after the initial 25-d culture, there was substantial swelling at the incision of the explant (Fig. 1b). A mixture of various types of calluses was formed when the twigs (approximately 15 d after germination, with buds) were cultured for 25 d as explants (Fig. 1c). However, the callus propagation was relatively slower than that of the twigs when the hard branches (the spring sprouting branches collected in October of the same year) were taken as explants and cultured for 25d (Fig. 1a). When the tender leaves were taken as explants in the initial culture to the 7th day, a reddish-brown, yellow, loose and green callus started at the edge of the blade. After the 25-d culture, the leaves began to curl from the edge to the petiole, forming multiple loose callus (Fig. 1d). The leaves were covered by a large number of callus after being sub-cultured, and the callus were mostly reddish-brown, green and yellowish curled. Compared to twigs and hard branch, the blade callus induction during the early stage was relatively slow, and the callus were mostly granular and loose. But in later stage, the callus proliferation rate of blade was significantly faster than that of twigs and hard branch, and showed a looser callus state than before.

There were 10 explant callus types in this stage. The type I callus (indicated by an arrow in Fig. 1a, I) was a white and loose callus that was fragile when there was successive transfer of the culture by explant induction on the lenticels. The induction of the adventitious roots stage can be directly induced from the callus. The type II callus (indicated by an arrow in Fig. 1a, II) appeared to grow longer in hard branches and was pale yellow and translucent with water damage. After successive generations, the callus turned brown and died. In the microscopic examination, the cells were fewer and more irregular in shape, and the cells were highly vacuolated. The type III callus (indicated by an arrow in Fig. 1b, III) appeared in the stem section and had a yellow, spherical, compact shape, with a rapid reproductive rate. The type IV callus (indicated by an arrow in Fig. 1b, IV) appeared in the twig stem section and was yellow-green and globular, multiplying faster and easily forming an embryonic callus. The type V callus (indicated by an arrow in Fig. 1c, V) was green with a spherical, compact shape. After successive transfers, the callus appeared similar to the type of callus that is white on the outside and pale yellow on the inside. The type VI callus (indicated by an arrow in Fig. 1c, VI) was bottle-green and globular, with the fastest147 reproduction rate, and its later form was not easy to change. In the microscopic examination, the cells were larger and elliptical, with dense cytoplasm, which was between the embryonal and non-embryonic cells. The type VII callus (indicated by an arrow in Fig. 1c, VII) was pale yellow and loose. After the subculture, this type browned and died. The type VIII callus (indicated by an arrow in Fig. 1c, VIII) was milk-white flaxen in a loose state and was more on the edge of the callus formation. After the subculture, the reproduction rate was slow and easily exhausted. The type IX callus (indicated by an arrow in Fig. 1d, IX) formed in the blade explants and had green water damage, and its callus cells died easily. The type X callus (indicated by an arrow in Fig. 1d, X) formed in the blade explants and was a green, dense granule, and it was a reddish-brown callus, although, at times, it did not induce embryonic callus formation.

**Fig. 2:**
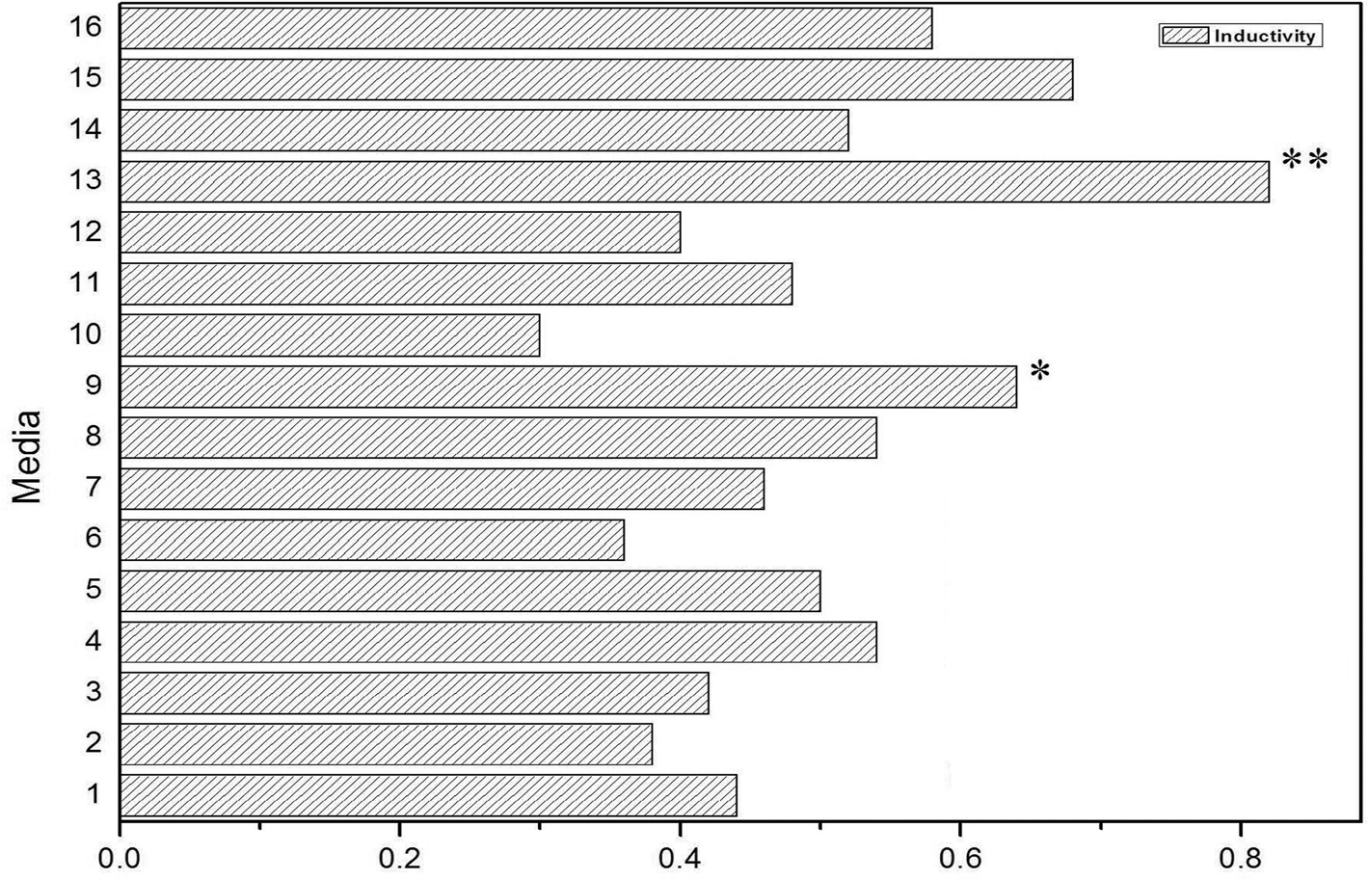
The influence of medium type and PGRs on callus induction

During the initial induction stage, the explants broke the normal propagation mode and built a new adaptive mechanism. Several experiments have shown that this mechanism is closely related to the type of explants, the type of culture medium and the type and content of PGR. The orthogonal experiment results showed that the callus induction rate in the MS + 0.8 mg/L TDZ + 1.0 mg/L 6-BA + 0.5 mg/L IAA medium was the highest, as high as 82% (Fig. 2). The effect of culture conditions on callus induction was TDZ > medium> IAA > 6-BA, the medium (*P* = 0.049) and TDZ (*P* = 0.037) had significant effects on callus induction (*P* < 0.05), while 6-BA (*P* = 0.17) and IAA (*P* = 0.338) had no significant effect (*P* > 0.05).

### Somatic embryo induction and somatic embryogenesis

**Fig. 3:**
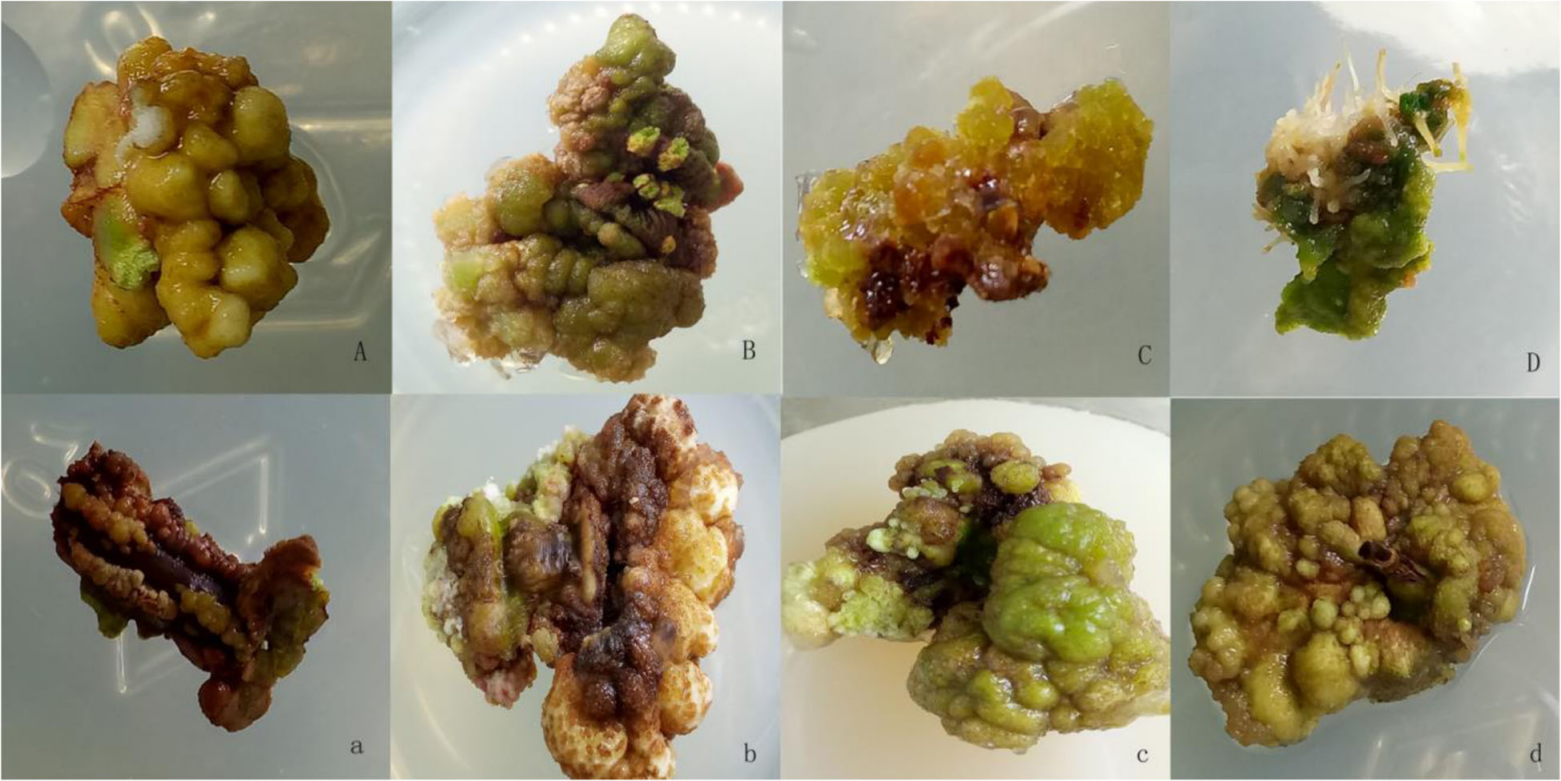
Somatic embryo induction and embryonic cell regeneration of embryonic callus A: After three generations, the green spherical callus was observed to contain a small number of embryonic cells. B: After five successive generations, the callus differentiated the embryonic callus cell population. C: The embryonic callus after 6 successive generations; D: After 7 generations, the somatic embryo matured and the callus showed polyembryonal stalks. a: The green callus that has not been sub-cultured in time in the initial culture gradually dried up and turned brown, and some cells even died; b: After being sub-cultured 25 d, white callus appears on the edge of brown callus. c: After two successive generations of 60 d, a dark green, spherically dense callus was formed. d: After 45 days of subculture, embryonic cells began to form on the callus edge

In the process of embryonic callus induction, type VI callus with faster reproduction rate and good growth was selected as the embryonic callus induction material. After a 56-d culture, the callus gradually changed into white somatic embryos (Fig. 3D). In the early stage, the surface embryo cells were increasing, but the proliferation rate was relatively slow (Fig. 3A-B). After the secondary 5-week culture, the callus was transferred to a medium without PGR. The microscopic examination revealed that the cells were small and elliptical, with dense cytoplasm, presenting typical embryonic cell characteristics (Fig. 3C). The process is achieved under PGR regulation, while embryogenesis is composed of proembryogenic masses (PEM), which may indicate that PEM formation is the normal reaction of the explants to PGR. Although it is difficult to strictly distinguish between direct or indirect somatic embryogenesis, embryogenesis is guaranteed to undergo somatic embryo formation when the PEM continues to develop and form an indeterminate organ (Fig. 3D). In this study, the somatic embryo was derived from the spherical tissue block, and this spherical tissue embryo was the equivalent cleavage polymorphism that formed the multicolumn embryonal cell embryo phenomenon presented in Fig. 3D. Unlike somatic embryos, meristematic tissues can be independently oriented to form roots or stems (which will also grow from within meristematic tissues), and somatic embryos grow almost without exception on the surface of callus tissue, and are easily separated from surrounding cells.

When the callus has not been sub-cultured for a long time, its surface gradually becomes dark brown. At this time, the embryogenic callus cell disappeared, and the callus was almost dead, which occurred from outside to inside (Fig. 3a). After the callus was cut, fit the longitudinal section to the surface of the MS + 2.0 mg/L TDZ + 1.0 mg/L 6-BA + 0.5 mg/L NAA + 35 g/L sucrose + 7.5 g/L AGAR semi-fixed medium for subculture. After a 25-d culture, the brown explants turned green (Fig. 3b). When this reactivated callus was further cultured, we found that the original healing surface appeared white with green and brown healing (Fig. 3b). When this callus continued to be inoculated in the embryonic induction medium of MS + 0.6 mg/L TDZ + 0.5 mg/L 6-BA + 2.0 mg/L IAA + 35 g/L sucrose + 7.5 g/L AGAR semi-fixed culture medium, the callus gradually changed to a green dense globule (Fig. 3c). On the surface of the callus, some cells were found to have embryonal cell traits (Fig. 3d).

### Adventitious bud induction

**Fig. 4:**
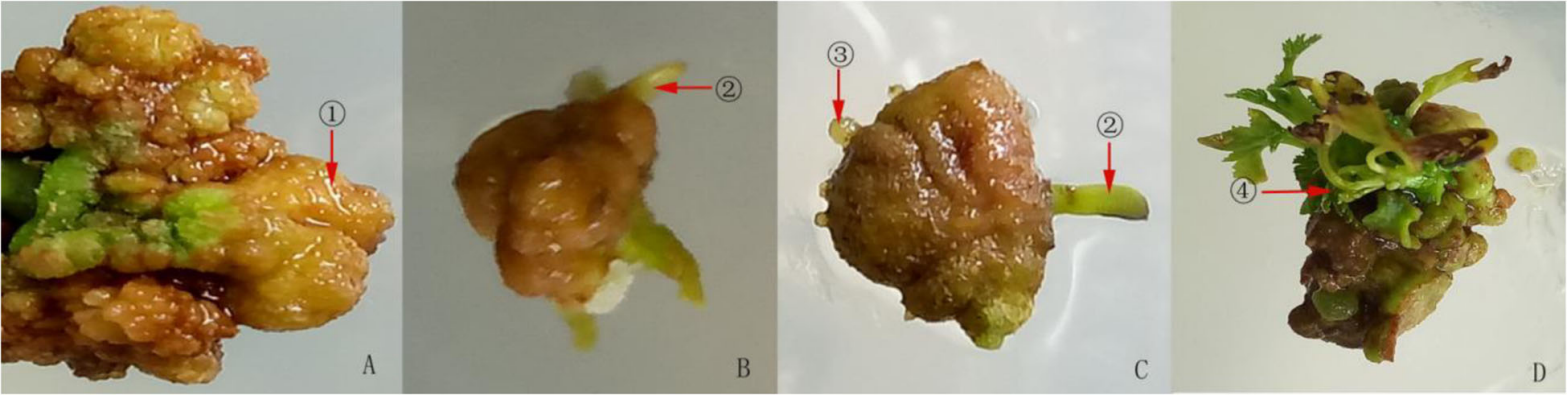
The adventitious bud induction of embryonic callus **a**: On the basis of mature embryo callus, the adventitious bud germinated after being cultured 7-9d. **b**: After 2 subcultures, the elongation of adventitious bud was increased; **c**: Continue to culture 30d, new adventitious buds formed like ③; **d**: After a 40d subculture, bud ② unfolded to form branches

When the mature embryogenic callus were cut and inoculated into the adventitious bud induction medium, the green adventitious bud gradually formed (Fig. 4a). After the subculture, embryonic callus began to swell, and the adventitious bud elongated (Fig. 4b). After 30 days of continuous cultivation, the adventitious buds begin to unfold (Fig. 4c). The stem branches were clearly visible at 40 d (Fig. 4d).

**Fig. 5:**
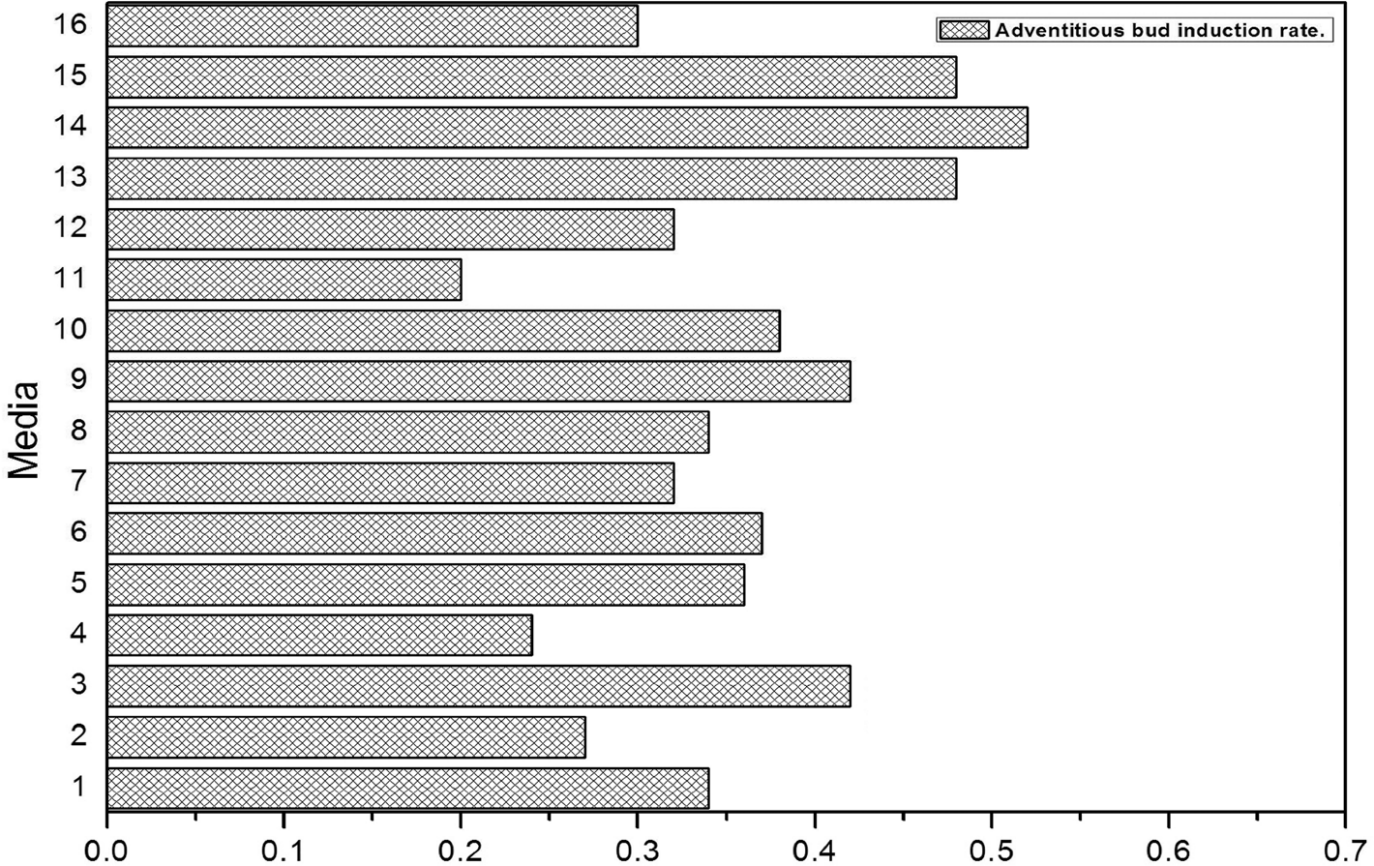
The influence of kinds and concentrations of different growth regulators on adventitious buds

It was difficult to distinguish between the inner embryonic cells and non-embryonic cells. However, embryonic cell orientation development is closely related to the in vitro culture environment and can be achieved by changing the in vitro culture conditions, especially PGRs. The results of the orthogonal test showed that when the exogenous growth regulator was 1.0 mg/L TDZ + 3.0 mg/L 6-BA + 0.2 mg/L NAA + 1.2 mg/L IAA, the adventitious bud induction rate was the highest (Fig. 4), reaching 52% (Fig. 5). The effect of different PGRs on adventitious bud induction rates was different, and the PGR influence sequence was 6-BA > IAA > TDZ > NAA. Moreover, 6-BA (*P* = 0.022), IAA (*P* = 0.027) and TDZ (*P* = 0.046) had the most significant effects on callus induction, and the impact of NAA (*P* = 0.0678) was not significant.

### Induction of adventitious root

**Fig. 6:**
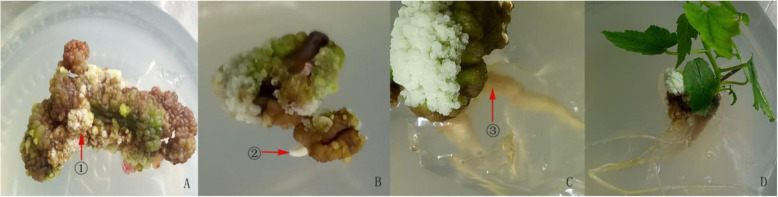
The adventitious root induction of embryonic callus **a**: After culturing type VI callus in adventitious root induction medium for 23 d, the embryonic callus gradually became white and loose and formed many infinitive roots. **b**: After 60 d of continuous cultivation, the adventitious roots of embryonic callus without adventitious buds germinated in large quantities. **c**: After the callus that had been differentiated to form adventitious buds was cultured for 25 days, the green embryonic callus gradually changed to white, and a large number of adventitious roots were generated on it. **d**: The amount of adventitious roots and leaves thrived after 20 d

Root induction is the last key link in the success of the rapid propagation of red maple, and the thickness and quantity of the adventitious roots affect the survival rate of the subsequent acclimation and transplants. When the embryonic callus type VI and embryonic callus with adventitious bud were inoculated on adventitious root induction medium and cultured for 15 d, the green embryonic callus gradually turned white and generated many adventitious roots (Fig. 6c). Furthermore, after cultivation for 20 d, the adventitious roots of the embryonic callus with buds germinated, and the plant leaves increased (Fig. 6d).

**Fig. 7:**
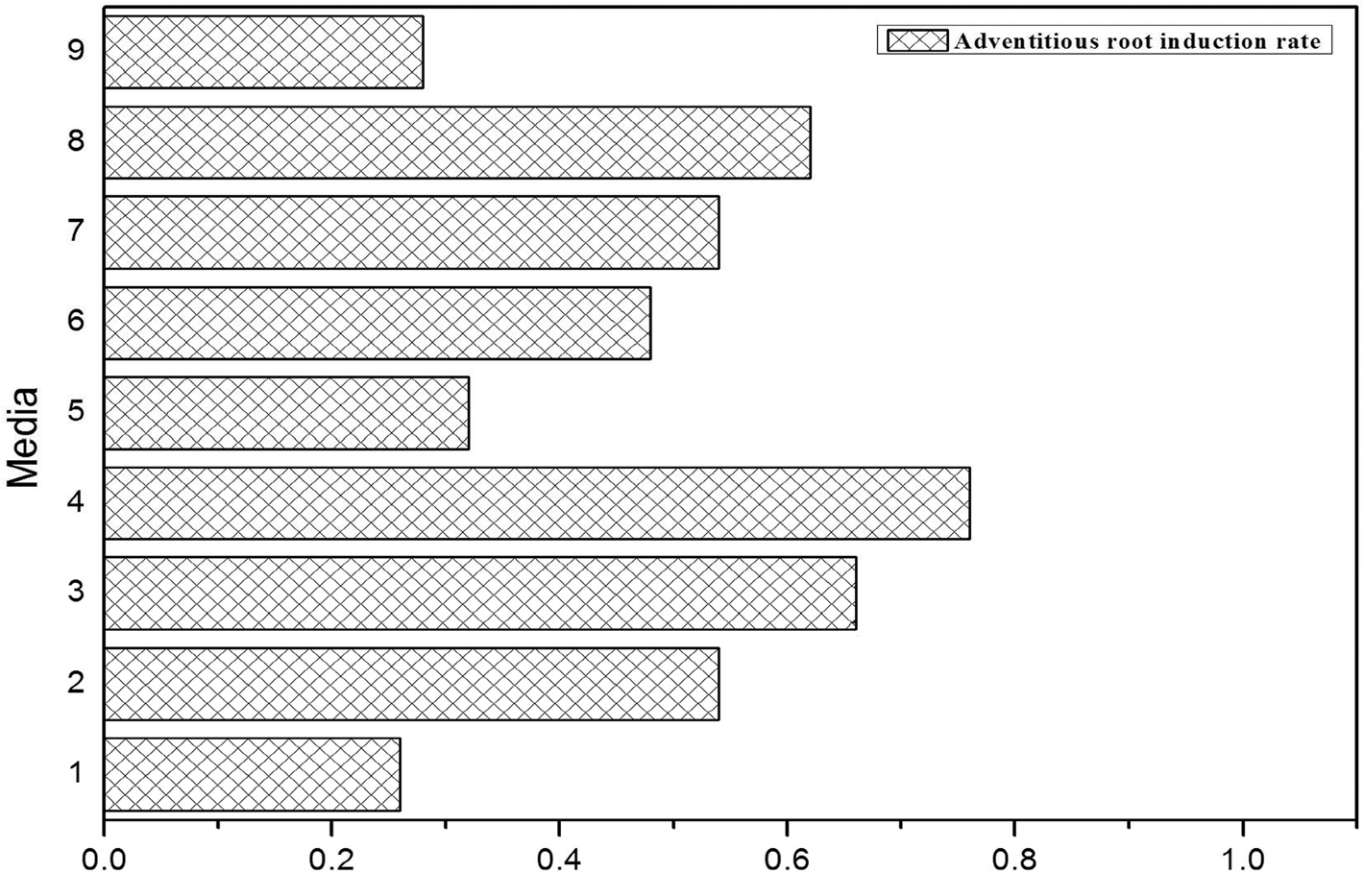
The influence of kinds and concentrations of different growth regulators on adventitious root

This study found that the adventitious roots can be induced directly from the stem and can also be induced through an embryonal callus (Fig. 6). The orthogonal experiment showed that the induction rate was as high as 76% (Fig. 7), when the MS + 0.6 mg/L TDZ + 1.0 mg/L 6-BA + 3 mg/L NAA + 35 g/L sucrose + 7.5 g/L AGAR semi-fixed medium were used. Moreover, with an increase in the NAA concentration, the induction rate of adventitious roots increased. The main effect of NAA on the induction of the adventitious roots process was the most significant (*P* = 0.023), while 6-BA (*P* = 0.442) and TDZ (*P* = 0.816) had no significant influence on the adventitious root process.

### Effects of different training methods on survival rate of plantlets

**Fig. 8:**
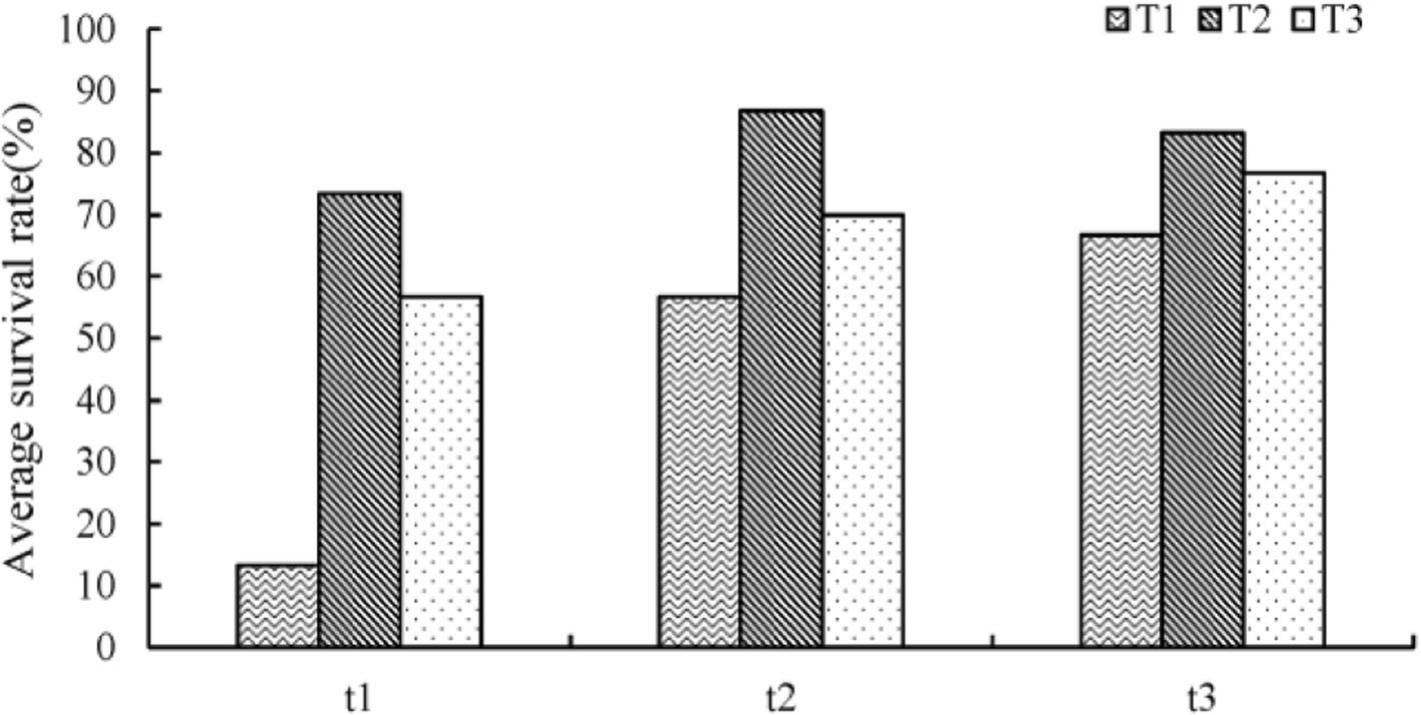
Effects of different training methods on survival rate of plantlets

Among the 9 treatments, it can be seen that the survival rate of T2t2 group (namely the group under 6-d culture in closed flasks and 2-d in opened flasks) was the highest, reaching 86.7% (Fig. 8). And the survival rate of transplanted plantlets increased first and then decreased with the increase of bottle closing time.

### Effects of different substrates on survival rate of transplanted plantlets

**Fig. 9:**
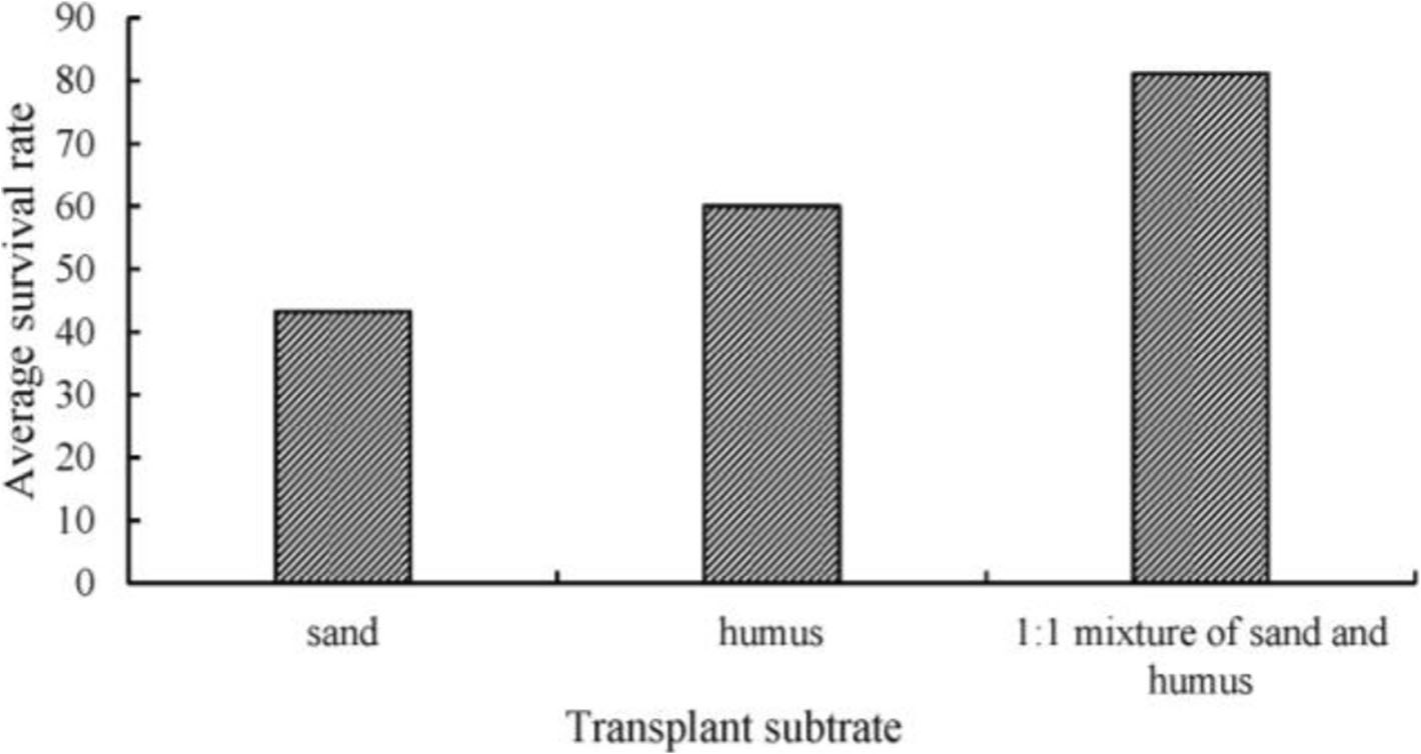
Effects of different substrates on survival rate of transplanted plantlets

The plantlets of T2t2 group were used as experimental materials. The survival rate of plantlets under different substrate treatments was significantly different, and the average survival rate of plantlets under 1:1 mixture of sand and humus treatment was the highest, reaching 81.1% (Fig. 9).

## Discussion

### The effect of PGRs in callus induction stage

Endogenous hormones naturally exist in plants; a low concentration of these compounds controls the whole process of cell division and elongation [19]. Hormones also play an important role in establishing and maintaining plant polarity [20], the most notable of which is the maintenance of apical dominance. In this study, we explored callus induction and embryo regeneration of the red maple. In the early stages of induction and propagation, the explants and callus were more dependent on PGRs, especially the cytokinin TDZ. As cytokinins TDZ was applied in a site, such as leaves, bark, evolution will make the processing units for a single amino acid active library. The surrounding amino acids were moved to the library, thereby promoting the synthesis of RNA and proteins and enhancing the activity of certain enzymes [21, 22]. This also explains the phenomenon in this experiment: when cytokinins, especially TDZ, were added to the medium, the apical dominance of the shoot segment with buds was inhibited, the lateral buds germinated into a single stem branch, and the proliferation of callus cells was promoted. In tissue culture (as well as intact plants and plant organs), cytokinin can also shorten the metaphase of cell division and thus accelerate cell division and proliferation [23]. Therefore, PGR plays an important role in callus induction and propagation.

### The regeneration of somatic embryos

Some embryonic cells can be formed gradually when the non-embryonic cells mutate through several divisions. If the secondary embryonic callus induction medium is present, embryonic callus can continue to form. In contrast, if cultured on the same medium, the embryonic cells will eventually be covered by non-embryonic cells, causing the embryonic callus to disappear. This result is illustrated by the phenomenon of callus degeneration after several successive generations of this experiment. In this experiment, it was found that when the culture medium, at the early stage of tissue culture, provides an adequate nutrition for the complete life cycle, the embryonic cells begin to multiply and differentiate into adventitious organs. If this supply is sustained, the number of cells will multiply in the “S” growth curve., but with the production of cells and the consumption of nutrients from the medium, the supply will continue to shrink and the cells will start to develop in the reverse direction, namely to develop into non-embryonic cells. This result is due to the restrictions of the culture medium space environment and nutrient supply [24]. Therefore, after the “S” curve reaches the highest point, the rate of cell proliferation will continue to decline, and the ecological niche of the cell population will be sharply constricted. Even the embryonic cells that have been formed will no longer have a survival advantage [25]. Then, the cellular self-protection mechanism is activated [26], and non-embryonic cells will cover the cell population. Therefore, it is thought that cell reproduction will continue to be carried out by successive generations before reaching the highest point of the “S” curve. In this way, the sustained proliferation of the cells can be maintained by the nourishing of the new culture medium.

The explanation is derived from “competition” [27], which is the disappearance of cellular embryos. After multiple cell divisions, if the medium cannot supply enough energy to maintain the normal division of embryonic cells, the cells will return to their best state of preservation, that is, the state of non-embryonic cells. We call this “the backward development of cells. This kind of self-protection mechanism will benefit the cell life cycle when there is a deteriorated environment [28]. If the environment changes again to the appropriate conditions, the embryonic cells will be activated again, and develop into new plants under suitable conditions.

This study explored the activation process of this browning callus. The results showed that during the 30-day culture without renewing the medium, the callus growth began to slow down on the 18th day. After cultivation for 24 d, the white and green callus gradually turned brown. After a culture of 40 d, the callus was mostly brown. After the brown callus were sub-cultured in the new medium, green and white callus gradually formed from the original explants at the 20d, and this time was significantly delayed compared with the initial induction time of the explant. This study also found that the callus induced from leaves was usually loose and light yellow, while that of twigs and branches was mostly white and green, and the formation of the former needed more time. This result indicates that the explants first break the original propagation mode before forming callus. Compared with twigs and branches, leaves have higher specialization process, thus their original propagation pattern is more difficult to break through.

### The formation of adventitious organs

Typically, every normal cell in a plant has the totipotency to develop into a complete plant but only in the best environment. In an appropriate medium, calluses can maintain long-term familiar stimulation and regenerate stems and branches, such as rice callus in 2% sucrose and 3% mannitol medium, and can maintain their root and embryo callus formation of bark for a long time [29]. Hormone, as the most effective factor in the induction of embryogenic callus, can play different roles under different combinations and proportions of hormone and the interaction with other factors. Abscisic acid (ABA), which is often used as a growth inhibitor in tissue culture, can significantly promote the maturation and germination of embryonic cells in tissue culture of some plants [30], and the addition of ABA to the medium can effectively promote the synthesis and accumulation of nutrients in embryonic cells [31]. In tissue culture of Saposhnikovia divaricata, normally differentiated embryonic cells can only be obtained by adding ABA [32]; For Persian walnuts, ABA promotes the maturation and germination of somatic embryos and the growth of both buds and roots [33–35]. When ABA is combined with other hormones or other factors such as cold treatment, the plant conversion rate can be significantly improved [36]. The formation of adventitious organs requires the combination of auxin and cytokinin, our results showed that when the ratio of NAA to TDZ was greater than 3:1, the bud induction mechanism was activated. The formation of adventitious buds is more complicated than that of adventitious roots. However, when the ratio increased to 5:1, the adventitious bud induction rate began to decrease. This result may be caused by the polar transport of auxin. If the callus was cultured in this kind of medium, it will lead to the death of embryonic cells [37]. In the process of low concentration to high concentration, its enhancement effect is increasing continuously. However, when reaching a certain threshold, higher concentrations of auxin also exhibit inhibitory effects [38] because most auxin has the physiological effect of the bell-shaped active curve [39]. In this study, auxin NAA was effectively absorbed by red maples, especially at 4 mg/L with a high root induction rate of 90%. Callus proliferation in the culture medium containing NAA was slow but the addition of NAA can induce the formation of adventitious roots. However, it is necessary to induce the adventitious root of the blade callus in the embryo callus stage. In this experiment, the rooting rate of explant was greater than that of the callus. Moreover, when the NAA concentration increased, the callus induced by stems was obviously stagnant. As a highly differentiated organ, the leaf cannot directly enter the rooting state and gradually become dry. Thus, it is more difficult to break the inherent propagation mechanisms during the process of inducing the formation of adventitious organs from highly developed plant organs.

## Conclusion

In this study, we screened the most suitable callus type for embryonic callus induction and selected suitable media formulations in various stages including callus induction, Somatic embryo induction and somatic embryogenesis, adventitious buds and adventitious root induction. In the course of our research, we found that PGRs play an important role in the callus induction stage, and the effect of TDZ is particularly obvious; The callus cells grow and proliferate according to the “S” growth curve, and can be sub-cultured when the highest growth point is reached to maintain the rapid proliferation of the callus cells and to avoid inactivation of callus caused by tight niche.

## Methods

### Source of plants

Plant materials were obtained from the forestry test base of Southwest University, and were formally identified by Associate Professor Liu Yu-min. The plants (Southwest University deposition number SWU-OG-03) are kept in the base (not a voucher specimen but the whole living plants) and can be obtained with the permission of Associate Professor Liu.

### Treatment and training conditions of test materials

Four types of materials were selected as explants in this experiment: tender leaves, twigs (approximately 15 d after germination) with buds, twigs without buds and hard branch (the spring sprouting branches collected in October of the same year). First, the explants were rinsed with tap water for 5 min, and then they were added to a 500 ml beaker. Then, the explants were soaked for 5 min in detergent water and washed with a hair brush, and surface stains were removed. Then, the explants were rinsed with tap water for 20 min, cut into 1–2 cm sections and moved to a sterile counter. The leaves were rinsed with a small flow of water, and all the injured and browning leaves were discarded when the surface was washed. The pretreated explants were disinfected with 75% alcohol on a sterile operating table for 30 s and treated with 0.1% HgCl_2_ for 8 min. The materials were rinsed 6 times with sterile water, and then the cutting sites were excised. The culture medium (PH5.8–6.0) used in the experiments was sterilized at 121 °C for 20 min;Hormones were sterilized by filtration.AD7000 digital camera (Nikon Corporation, Tokyo, Japan) using manual settings (F = 4.5, shutter 320 s, iso = 500) was used to photograph the callus regularly and observe and record the changes.

### The induction of callus

An orthogonal experiment was designed with medium (DKW, N6, MS,/ WPM), and the growth regulator IAA (0.5, 1.0, 1.5, 2.0 mg/L),6-BA (0.5, 1.0, 1.5, 2.0 mg/L) and TDZ (0.2, 0.4, 0.6, 0.8 mg/L), with a total of 16 treatments (Fig. 2). Each group was treated with 10 bottles of 5 to 6 explants. The culture bottles were placed in an artificial climate box (RGX-250) and cultured under conditions of humidity 75%, 25 °C, 14 h of light (1500 lx) and 10 h of darkness. The changes of explants were observed and recorded every 2 days.

### Embryo callus induction and somatic embryogenesis

During the stages of callus induction and proliferation, the MS + 0.6 mg/L TDZ + 0.5 mg/L 6-BA + 2.0 mg/L IAA + 35 g/L sucrose + 7.5 g/L AGAR semi-fixed culture medium effectively induced the formation of somatic embryo, and the somatic embryo induction rate was as high as 41 per gram. Based on the above results, the light yellow and white, loose callus was selected to induct somatic embryo formation in the medium (pH = 5.8). After 5 weeks, the somatic embryo was transferred to the MS medium free of growth regulating substances and cultured until the embryo matured. Culture conditions were 85% humidity, (25 + 2) °C, 16 h of light (1500 lx) and 8 h of darkness. During the culture, the changes of callus and somatic embryo were observed and recorded once every 2 days.

### Induction and germination of adventitious bud

To obtain adventitious buds, the embryonic callus was used as the experimental material, and an orthogonal experiment was designed with TDZ (0.5, 1.0, 1.5, 2.0 mg/L), 6-BA (1.0, 2.0, 2.5, 3.0 mg/L), NAA (0, 0.1, 0.2, 0.5 mg/L),and IAA (0.6, 0.8, 1.0, 1.2 mg/L), with a total of 16 treatments (Fig. 5). Culture conditions were 85% humidity, (25 + 2) °C, 18 h of light (3000 lx) and 2 h of darkness (20 h cycle). During the culture, the changes of callus and the germination of adventitious bud were observed and recorded once every 2 days.

### The induction and germination of the adventitious root

Based on the above test results, the obtained embryonic callus and embryonic callus with buds were used as experimental materials for adventitious root induction. An orthogonal experiment was designed with NAA (1.0, 2.0, 3.0 mg/L), 6-BA (0.5, 1.0, 2.0 mg/L), and TDZ (0.6, 0.8, 1 mg/L), with a total of 9 treatments (Fig. 5). Culture conditions were 85% humidity, (25 + 2) °C, 12 h of light (1500 lx) and 12 h of darkness. During the culture, the changes of callus and the germination of adventitious root were observed and recorded once every 2 days.

### Training of plantlets

After the plantlets were moved to the nursery room at room temperature of 20 °C, they were first cultivated in closed flasks for T1 = 3d, T2 = 6, T3 = 12d respectively, and then the lids were opened to continue cultivating for t1 = 1d, t2 = 2d, t3 = 3d, with a total of 9 treatments, 30 plantlets per treatment. After the cultivation, the plants were transplanted, and the survival rate was counted 30 days after transplanting.

### Screening of transplanting substrate

The plantlets obtained from the best training method were transplanted into sand, humus soil, and a 1:1 mixture of sand and humus respectively, and the survival rate was counted 30 days after transplanting.

### Data statistics and analysis

The callus induction rate (%) = callus/explants number × 100%.

The somatic embryo induction rate (%) = the number of somatic embryo/callus mass × 100%.

The adventitious bud germination rate (%) = the number of adventitious bud/ the total number of callus × 100%.

The adventitious root germination rate (%) = the number of adventitious root/ the total number of callus × 100%.

The average survival rate (%) = the number of viable plantlets/ the total number of plantlets × 100%.

The data was analyzed using SPSS version 20.0, and Duncan’s new multiple range test was used for multiple comparisons.

## Data Availability

The data used to support the findings of this study are available from the corresponding author upon request.

## Abbreviations

2, 4-D: 2, 4-Dichlorophenoxyacetic acid
6-BA: 6-Benzylaminopurine
ABA: Abscisic acid
DKW: Driver and Kuniyuki walnut medium
IAA: Indole-3-acetic acid
IBA: Indole-3-Butytric acid
MS: Murashige & Skoog
N6: Chu’s N-6 Medium
NAA: 1-Naphthaleneacetic acid
PGRs: Plant growth regulators
TDZ: Thidiazuron
WPM: Woody Plant medium
